# Using In Situ Measurements to Experimentally Characterize TiO_2_ Nanoparticle Synthesis in a Turbulent Isopropyl Alcohol Flame

**DOI:** 10.3390/ma14227083

**Published:** 2021-11-22

**Authors:** Benedetta Franzelli, Philippe Scouflaire, Nasser Darabiha

**Affiliations:** Laboratoire EM2C, Université Paris-Saclay, CNRS, CentraleSupélec, 8-10 rue du Joliot Curie, 91190 Gif-sur-Yvette, France; philippe.scouflaire@centralesupelec.fr (P.S.); nasser.darabiha@centralesupelec.fr (N.D.)

**Keywords:** in situ optical diagnostics, flame synthesis, TiO2, turbulence

## Abstract

The objective of the present work is to show the potential of in situ measurements for the investigation of nanoparticles production in turbulent spray flames. This is achieved by considering multiple diagnostics to characterize the liquid break-up, the reactive flow and the particles production in a spray burner for TiO${}_{2}$ nanoparticle synthesis. The considered liquid fuel is a solution of isopropyl alcohol and titanium tetraisopropoxide (TTIP) precursor. Measurements show that shadowgraphy can be used to simultaneously localize spray and nanoparticles, light scattering allows to characterize the TiO${}_{2}$ nanoparticles distribution in the flame central plane, and spontaneous CH* and OH* chemiluminescences, as well as global light emission results, can be used to visualize the reactive flow patterns that may differ with and without injection of TTIP. Concerning the liquid, it is observed that it is localized in a small region close to the injector nozzle where it is dispersed by the oxygen flow resulting in droplets. The liquid droplets rapidly evaporate and TTIP is quasi-immediately converted to TiO${}_{2}$ nanoparticles. Finally, results show high interactions between nanoparticles and the turbulent eddies.

## 1. Introduction

Synthesis in turbulent spray flames is today considered as a valuable alternative for large-scale production of nanoparticles with a relatively low cost. Laboratory-scale spray flame reactors [1,2,3,4,5,6,7,8,9,10,11,12] were therefore developed to improve our understanding of nanoparticles production in these reactive flows in order to better control the characteristics and properties of the final product. For this, ex situ measurements are classically performed to characterize the collected materials in terms of morphology, physical and optical properties depending, for example, on operating conditions such as temperature, pressure, and precursor concentration. However, it is expected that the properties of nanoparticles produced via flame synthesis will depend on the experienced local conditions governed by the flow and the flame. Therefore, it would be of interest to combine classical ex situ measurements to in situ optical diagnostics classically used in combustion research to understand the physical processes occurring during the flame synthesis by characterizing the spatial and temporal evolution of spray, flow, flame and nanoparticles. In addition, in situ measurements will allow the characterization of boundary conditions necessary to perform numerical simulations [13,14,15,16,17] and they provide an experimental database for their validation [9,18].

In this framework, the present study aims to prove the feasibility and the great interest of in situ measurements when investigating nanoparticles production in a laboratory-scale spray flame reactor. The burner consists of a spray nozzle where the liquid fuel is atomized by an annular flow of oxygen, a circular pilot premixed ethylene/air flame and a coflow of pure N${}_{2}$. The considered liquid fuel is a solution of isopropyl alcohol and titanium tetraisopropoxide (TTIP) precursor.

Three main processes characterize the flame: (a) the break-up of the liquid jet, (b) the turbulent reactive flow, and (c) the production of the particles. The spatial localization of these three processes is here provided using in situ experimental measurements, classically used in combustion research: (a) shadowgraphy and light scattering for the liquid phase, (b) flame luminosity, CH* and OH* chemiluminescences to characterize the combustion process, and (c) light scattering to localize TiO${}_{2}$ nanoparticles.

The paper is organized as follows. First, the experimental setup is described by presenting the flame synthesis burner and the different optical diagnostics considered in this work. Then, the potentials and the difficulties in applying shadowgraphy and light scattering to the characterization of nanoparticles flame synthesis are discussed. Finally, results are presented by looking at the three different processes that characterize nanoparticles’ flame synthesis.

## 2. Experimental Setup

### 2.1. Flame Synthesis Burner

**Figure 1:**
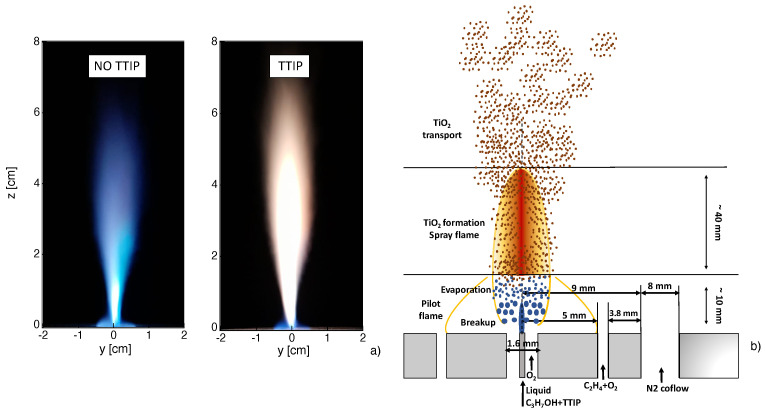
(**a**) Flame luminosity without and with injection of TTIP. (**b**) Schematic presentations of the reactor inlet and of the processes inside the reactor.

The burner (ParteQ GMBH model LS-FSR, Malsch, Germany) studied in the present work is the same as used in [2,3,4,5,15]. The burner, schematically presented in Figure 1b, allows the stabilization of a turbulent spray flame, whose luminosity is visualized in Figure 1a. For this, liquid isopropyl alcohol (Sigma Aldrich, Saint-Louis, MO, USA, C${}_{3}$H${}_{8}$O) is injected through a syringe in the center. The liquid flow is provided by a Tuthill pump upstream of a mini-Coriolis flowmeter from Bronkhorst. The imposed flow rate is 0.005 Nl/min. The liquid jet is surrounded and dispersed by an annular jet of pure oxygen with a flowrate of 3 Nl/min. A premixed methane-oxygen pilot flame with an equivalence ratio of Φ=0.83 (oxygen flowrate of 1.2 Nl/min and methane flowrate of 0.5 Nl/min) is needed to stabilize the non-premixed flame. The coflow consists of pure nitrogen with a flowrate of 4 Nl/min.

The obtained flame is visualized in Figure 1a. As already observed in [15], the flame is not perfectly axis-symmetric mainly due to a non-symmetric pilot flame as a result of some geometrical imperfections of the burner. To consider TiO${}_{2}$ nanoparticles production, titanium tetraisopropoxide (TTIP), Ti(OCH(CH${}_{3}$)${}_{2}$)${}_{4}$, (Sigma Aldrich) with a purity of 97% is added to liquid flow in a proportion of 5 ml of TTIP for one liter of isopropyl alcohol. The obtained flame is visualized in Figure 1a. It can be observed that a more luminous flame is obtained when considering injection of TTIP.

### 2.2. In Situ Optical Diagnostics

**Figure 2:**
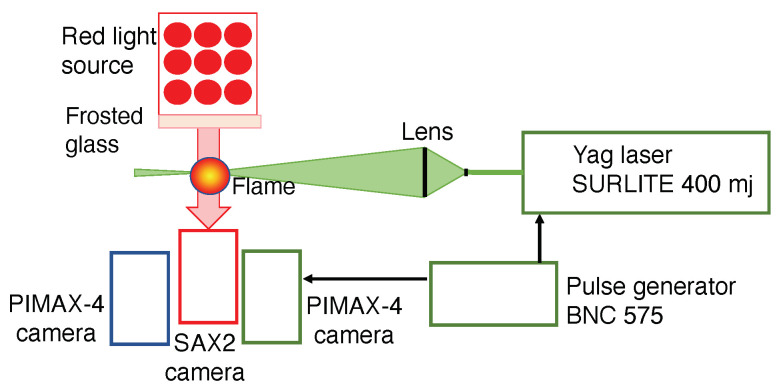
Schematic presentation of the optical setup.

The optical setup used to perform shadowgraphy, light scattering and emission measurements is schematically presented in Figure 2. The shadowgraph measurements are performed using a red backlighting system featuring a red LED spot. The red LED spot consists of a 7 cm × 9 cm rectangle of LEDs emitting at a dominant wavelength of 633 nm. A frosted glass is placed between the spot and the flame to get a light as homogeneous in space as possible. The spot is fed by a direct current power supply in order to avoid main current frequency interference. A Fastcam SAX2 camera (Photron, San Diego, CA, USA) is placed at the opposite side of the LED spot to obtain shadowgraphy images of the flame. It is equipped with a Nikon 105 mm f/2.8 lens and a 20 mm extension ring. Images of 1024 × 1024 pixels are obtained with a resolution of 62 μm/pixel. The signal acquisition gate width is 100 μs.

A YAG Surelite 400 mJ Continuum laser at 532 nm wavelength is used for the light scattering on the solid particles. A set of two lenses creates a laser sheet of 70 mm height and about 300 μm thickness, which passes through the burner central axis. The scattered light is captured by a PIMAX4 camera (Teledyne Princeton Instruments, Trenton, NJ, USA) (1024 × 1024 pixels) equipped with a Nikon lens 100F/1.8 and a 20 mm extension ring. It has a spatial resolution of 62 μm/pixel. A FF01 530 nm FWHM 11 nm filter (Semrock, Rochester, NY, USA) allows the observation of only light scattering. Both the camera and the laser are synchronized via a pulse generator BNC575 (Berkeley Nucleonics Corp, San Rafael, CA, USA). The images are captured with no delay and a gate width of 15 ns.

A second Teledyne Princeton PIMAX camera equipped with a Sodern UV lens 100F/2.8 is used for measurements of flame global spontaneous emission as well as CH* and OH* emissions. The spatial resolution is 95 μm/pixel. For flame global spontaneous emission, no filter is used on the camera and the exposure time is adjusted in order to not saturate the gray levels (5 μs). Regarding the OH* spontaneous emission, an Asahi 310 nm filter (96SA02), FWHM 10.00 nm is used in front of the camera lens. The exposure time is 30 μs. For CH* spontaneous emission, an Asahi 430 nm filter (F0102), FWHM 10.00 nm filter is used and an exposure time of 15 μs is retained.

Time-averaged results for all measurements are obtained by subtracting the background and considering 500 images.

## 3. Using Shadowgraphy and Light Scattering Diagnostics to Characterize Flame Synthesis

**Figure 3:**
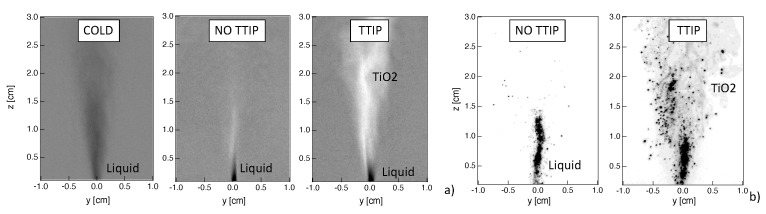
Instantaneous images obtained by (**a**) shadowgraphy and (**b**) light scattering, without and with injection of TTIP. Results for the non-reacting cold case with shadowgraphy are also presented.

Several phenomena can be visualized using the shadowgraphy measurements as illustrated in Figure 3a. First of all, when considering the non-reacting cold case, the presence of the liquid jet and of the spray can be detected since the objects between the light source and the camera appear the darker the more they absorb the light. Similarly, the presence of liquid jet is detected in both reacting cases without and with injection of TTIP. It can be observed that the liquid phase occupies a smaller region, compared to the cold case, due to its quick evaporation due to high flame temperature.

In the reactive case, with the injection of TTIP, spots of light due to diffraction of partially transparent TiO${}_{2}$ nanoparticles can also be observed. Then, it is possible to discriminate spray from TiO${}_{2}$ nanoparticles by considering dark or bright information. Therefore, thanks to the shadowgraphy, it is possible to get simultaneous information on the localization of both spray and of TiO${}_{2}$ nanoparticles.

Since the shadography measurements provide line-of-sight-integrated information, light scattering measurements were also performed to investigate spray and TiO${}_{2}$ nanoparticles distribution at the burner central plane. Instantaneous images for both cases with and without injection of TTIP are presented in Figure 3b. Although planar information can be obtained with this technique, however, a rigorous discrimination between the signals from spray and TiO${}_{2}$ nanoparticles is not straightforward. It has been observed that, in the region close to the liquid injection, the contribution from the liquid spray scattering is predominant compared to the one from nanoparticles. Therefore, it is assumed in the following that, in the spray zone close to the burner, a high intensity signal corresponds to spray light scattering, whereas low intensity corresponds to TiO${}_{2}$ nanoparticles. Even if such criterion is arbitrary, the complementary use of shadowgraphy allows to identify the region where the liquid phase is expected to be observed (in our case for a height above the burner z<2 cm). Indeed in this region, results from light scattering should be analyzed with caution, but beyond this region, light scattering information can be used to localize the presence of TiO${}_{2}$ particles.

## 4. Characterization of Spray Flame Synthesis

The main processes occurring during the flame synthesis, schematically presented in Figure 1b, are described here thanks to in situ optical diagnostics, classically used in combustion research, comparing results on flames with and without injection of TTIP.

### 4.1. Liquid Injection and Spray

Thanks to shadowgraphy and light scattering measurements, the presence of liquid can be investigated. When looking at the instantaneous results of Figure 3, the presence of a central liquid jet core is observed. The liquid is localized in a small region close to the injector nozzle due to the effect of the dispersion oxygen flow, which leads to the break up of the liquid jet into droplets, together with the effect of the flame high temperature, which results in a rapid evaporation. Occasionally, big droplets can be observed at higher heights above the burner.

It should be noted that some differences are observed between the two techniques. In particular, a dense cylindrical liquid jet seems to be detected by light scattering whereas the atomization seems to occur more rapidly from shadowgraphy results. However, it has to be reminded that the two systems neither present the same sensitivity nor the same resolution of the liquid structures. Moreover, line-of-sight-integrated measurements are provided by shadowgraphy, whereas light scattering gives access to planar information. Finally, light scattering results may be affected by the fact that both spray and TiO${}_{2}$ nanoparticles are simultaneously detected.

**Figure 4:**
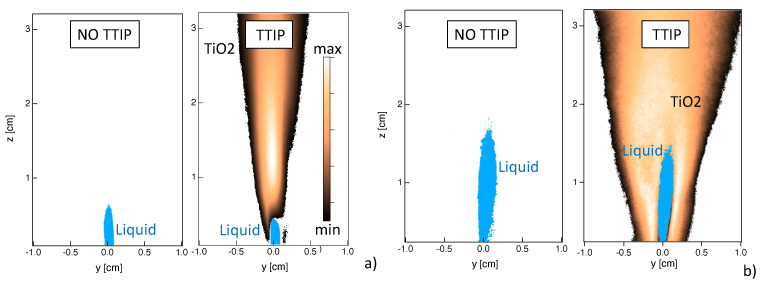
Localization of liquid phase (blue color) and TiO${}_{2}$ nanoparticles (brown palette) via (**a**) shadowgraphy, and (**b**) light scattering. Time-averaged results (left) without and (right) with injection of TTIP are presented.

Time-averaged shadowgraphy and light scattering results are presented in Figure 4 for the flames without and with injection of TTIP. A slightly shorter spray region is identified in the case of the flame with TTIP. However, no other significant differences are observed so that it can be deduced that the spray region is correctly identified by the light scattering technique even in the presence of nanoparticles. Therefore, the differences between results from light scattering and shadowgraphy are most probably due to the intrinsic specificity of these two techniques.

Even if a more detailed characterization of the performances of these techniques in the context of flame synthesis is desirable, some common conclusions on the spray process can already be drawn. First, high fluctuations of the spray position are observed (not shown). Second, results are not symmetric, possibly due to the difficulty in obtaining a perfect centering of the liquid injection syringe in the dispersion system. Third, TiO${}_{2}$ nanoparticles are formed close the spray, indicating that the nanoparticles production is an extremely fast process occurring once the TTIP precursor has evaporated. Finally, it can be said that the spray is not likely to be found for z>2 cm, so that in this region light scattering from spray can be considered as negligible compared to nanoparticles contribution. Therefore, results on the localization of TiO${}_{2}$ nanoparticles can be considered with confidence for z>2 cm.

### 4.2. Flame

**Figure 5:**
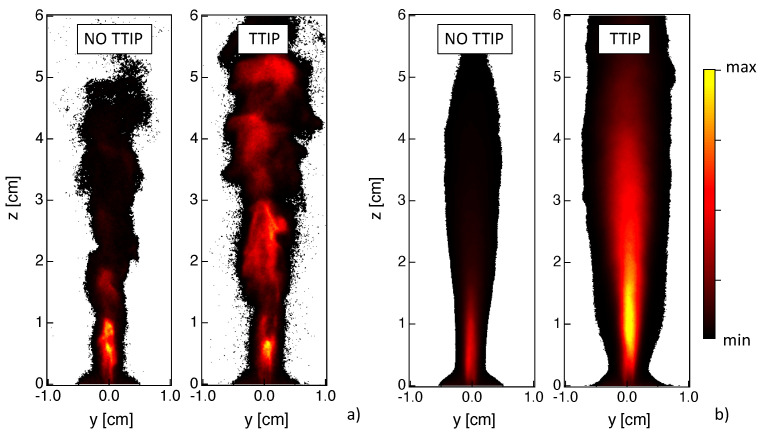
(**a**) Instantaneous and (**b**) time-averaged images of line-of-sight-integrated global emission for the reactive cases (left) without and (right) with injection of TTIP.

**Figure 6:**
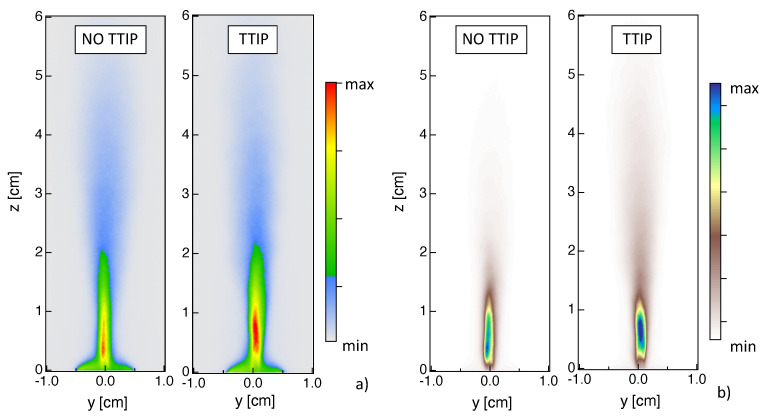
Time-averaged line-of-sight-integrated of (**a**) OH* and (**b**) CH* emission for the reactive cases (left) without and (right) with injection of TTIP.

The combustion process is investigated here by analyzing images of flame global spontaneous emission (Figure 5) as well as CH* and OH* chemiluminescences (Figure 6). The global spontaneous emission from the flame contains information on the whole flame emission, while CH* and OH* chemiluminescences can be used to localize the heat release zone. In the presented case, the signal from global spontaneous emission is generally 10 times more intense than the one from OH* and CH*.

Looking at the instantaneous global emission results, Figure 5a, a turbulent flame structure can be recognized even if these measurements provide line-of-sight-integrated information. When considering time-averaged results, Figure 5b, it can be noticed that the symmetry of the fields is not perfect, similarly to the results for spray in Figure 3.

Results are quite different between the two cases with and without injection of TTIP, indicating that the addition of TTIP has a non-negligible effect on global spontaneous emission, as already deduced from Figure 1a. When looking at the case without TTIP, the most relevant emission contribution due to the isopropyl flame is located along the central line at a small height above the burner (z<1 cm). The pilot flame is identified by the small conical emission region localized close to the burner tip and it only slightly contributes to the flame emission. In the case with injection of TTIP (Figure 5b), the maximum values of emission are found far above the burner (0.5 cm <z<1.5 cm) due to the presence of nanoparticles. In this case, spontaneous emission is the result of both flame and nanoparticles emissions. The maximum values of spontaneous emission for the case with injection of TTIP are higher by a factor of 10, compared to the case without injection of TTIP.

Time-averaged fields of OH* and CH* chemiluminescences are presented in Figure 6. Concerning results without injection of TTIP, CH* and OH* chemiluminescence signals present a similar spatial evolution compared to global emission. Most of the heat is expected to be released close to the region where spray evaporation occurs (z<2 cm), with a maximum located at z<1 cm. Post-combustion processes are observed up to z≈ 5 cm. Close to the injector, a lower signal intensity is measured compared to the central region, possibly indicating that the pilot flame only slightly contributes to the global heat release even if it is essential for the flame stabilization. Results with injection of TTIP are qualitatively similar to those without TTIP, even if more intense signals are observed for the TTIP case. This is probably due to the fact that the addition of TTIP leads to an increase of carbon atoms compared to a pure isopropyl alcohol flame. By comparing these results with those on global emission for the TTIP case in Figure 5, it can be confirmed that the maximum of global emission at z>1 cm is due to the presence of particles.

**Figure 7:**
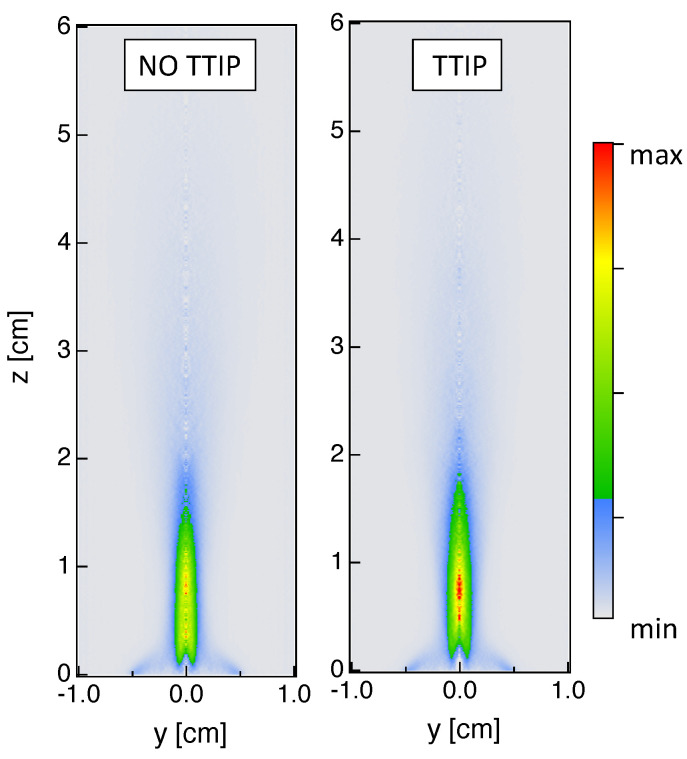
Planar information are extracted using the Abel-inversion on time-averaged results from OH* chemiluminescence for the reactive cases (**left**) without and (**right**) with injection of TTIP.

Classically, time-averaged line-of-sight integrated results could be transformed using an Abel-inversion to obtain 2-D planar information. Since the investigated flame is not quite axis-symmetric, as can be deduced by looking for example at the CH* results in Figure 6b, caution should be paid when analyzing Abel-inverted results. An example of planar results is presented in Figure 7 considering only the right half-side of the time-averaged results from OH* chemiluminescence. When looking at 2-D planar fields, a lower signal intensity is measured close to the injector compared to the central region. Therefore, it is quite evident by looking at 2-D fields that the pilot flame only slightly contributes to the global heat release even if it is essential for the flame stabilization. Globally, similar conclusions can be deduced compared to line-of-sight integrated results in terms of localization of maximum value of OH* signal and effect of TTIP addition on OH* emission.

### 4.3. Particles Production

**Figure 8:**
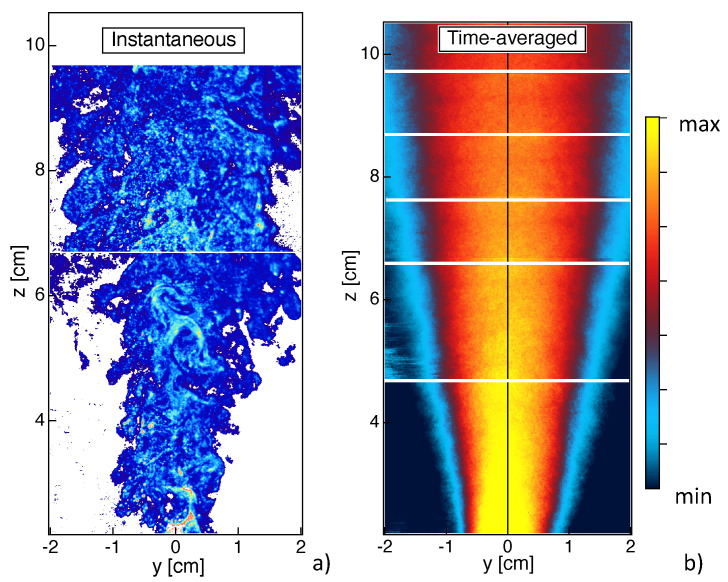
Localization of TiO${}_{2}$ nanoparticles via light scattering measurements. Instantaneous (**a**) and time-averaged (**b**) fields. Time-averaged field is reconstructed by assembling series of measurements at six vertical positions of the laser sheet.

Figure 8 presents light scattering measurements of TiO${}_{2}$ nanoparticles by gathering random collections of data at different vertical positions above the burner. As mentioned above, the presence of TiO${}_{2}$ nanoparticles can be analyzed by looking at both shadowgraphy and light scattering fields. When looking to results close to the injector (Figure 4), it can be seen that nanoparticles appear in the close proximity of the spray. This indicates that, once the liquid TTIP precursor has evaporated, it is rapidly converted into solid TiO${}_{2}$ particles, confirming that TiO${}_{2}$ production is governed by fast reactions. Once the particles formed, their localization seems to be strongly governed by turbulent eddies, as it can be observed from the instantaneous results of light scattering close to the injector (Figure 4b) and along the flame (Figure 8a). Then, TiO${}_{2}$ particles mainly concentrate along thin ligaments that are stretched and deformed by the turbulent flow eddies and are finally convected downstream the combustion region (z>4 cm).

Far downstream of the burner (z>7 cm), a more homogeneous spatial distribution of TiO${}_{2}$ particles is observed due to turbulent mixing. Time-averaged results of light scattering are presented in Figure 8b. A very intense signal is observed close to the burner, which decreases downstream the post-flame region. This high light scattering region seems to coincide with the high flame luminosity zone in Figure 1a. The light scattering signal depends on particle size and number density. Since the size of the particles is not expected to decrease along the height above the burner, the light scattering field seems to indicate that the nanoparticles are generated close to the spray region in large numbers and that the particles number density subsequently decreases due to collisional processes. Even if the distribution of the light scattering signal is not symmetric close to the burner (Figure 4b) due a non axis-symmetric flame, its time-averaged distribution becomes rapidly symmetric, probably due to the turbulent transport of the particles downstream.

## 5. Conclusions

The objective of the present work was to demonstrate the interest in in situ optical diagnostics classically used in combustion research for the investigation of TiO${}_{2}$ nanoparticles synthesis in turbulent spray flames. Shadowgraphy, light scattering, and global flame luminosity as well as CH* and OH* chemiluminescence measurements were employed in order to study the three main processes that characterize the spray flame. In this way, the liquid break-up, the reactive flow and the TiO${}_{2}$ nanoparticles production were analyzed.

Shadowgraphy measurements showed that it was possible to simultaneously localize the liquid phase and the nanoparticles. Light scattering results allowed to characterize the TiO${}_{2}$ nanoparticles distribution in the flame central plane. The liquid flow is localized in a small region close to the injector nozzle where it is dispersed by the oxygen flow resulting in droplets. The liquid droplets rapidly evaporate due to the high temperature of the flame. When TTIP is added to the liquid flow, right after its evaporation and due to its high reactivity, it is immediately converted to TiO${}_{2}$ nanoparticles. Global spontaneous emission is quite different when considering TTIP compared to the flame without TTIP. In specific, when TTIP is added, maximum emissions are observed far above the burner showing the non-negligible contribution of TiO${}_{2}$ particles emissions. On the contrary, even if CH* and OH* chemiluminescence signals are more intense when adding TTIP, the signals are qualitatively in agreement with the flame without TTIP. Finally, shadowgraphy and light scattering results at different heights above the burner showed high interactions between nanoparticles and the turbulent eddies. Even if in future works an optimization of these techniques to flame synthesis is desirable, in situ optical diagnostics from combustion research can be used to provide a new insight on flame synthesis, complementary to ex situ measurements.

## Data Availability

Data are available upon request to authors.
